# Research progress of engineered mesenchymal stem cells and their derived exosomes and their application in autoimmune/inflammatory diseases

**DOI:** 10.1186/s13287-023-03295-7

**Published:** 2023-04-11

**Authors:** Xueqing Zhu, Dan Ma, Baoqi Yang, Qi An, Jingwen Zhao, Xinnan Gao, Liyun Zhang

**Affiliations:** 1 School of Basic Medicine, Shanxi Medical University, Taiyuan, China; 2 Third Hospital of Shanxi Medical University, Shanxi Bethune Hospital, Shanxi Academy of Medical Sciences, Tongji Shanxi Hospital, Taiyuan, 030032 China

**Keywords:** Mesenchymal stem cells, Exosomes, Genetic engineering, Surface modification, Tissue engineering, Autoimmune diseases

## Abstract

Autoimmune/inflammatory diseases affect many people and are an important cause of global incidence and mortality. Mesenchymal stem cells (MSCs) have low immunogenicity, immune regulation, multidifferentiation and other biological characteristics, play an important role in tissue repair and immune regulation and are widely used in the research and treatment of autoimmune/inflammatory diseases. In addition, MSCs can secrete extracellular vesicles with lipid bilayer structures under resting or activated conditions, including exosomes, microparticles and apoptotic bodies. Among them, exosomes, as the most important component of extracellular vesicles, can function as parent MSCs. Although MSCs and their exosomes have the characteristics of immune regulation and homing, engineering these cells or vesicles through various technical means, such as genetic engineering, surface modification and tissue engineering, can further improve their homing and other congenital characteristics, make them specifically target specific tissues or organs, and improve their therapeutic effect. This article reviews the advanced technology of engineering MSCs or MSC-derived exosomes and its application in some autoimmune/inflammatory diseases by searching the literature published in recent years at home and abroad.

## Introduction

Autoimmune/inflammatory diseases, including rheumatoid arthritis (RA), systemic lupus erythematosus (SLE), osteoarthritis (OA), and inflammatory bowel disease (IBD), affect many people and are an important cause of global incidence and mortality[1, 2]. The causes and clinical manifestations of different autoimmune diseases are different, but most of them are recurrent and chronic persistent, which seriously affects the quality of work and life of patients. There is no ideal treatment for autoimmune diseases, and current treatment options include glucocorticoids, immunosuppressants, and some new biological agents [3–6]. Immunomodulatory drugs such as glucocorticoids and immunosuppressants have broad effects rather than disease-specific effects and thus may lead to side effects such as infection and malignancy [7]. Novel biologics employ targeted immunotherapy to inhibit major proinflammatory signals by blocking inflammatory cytokines (TNF, IL-6, etc.), cell surface molecules (B cells, T cells), and intracellular kinases (JAKs, etc.) Access [7, 8]. Its main characteristics are rapid onset of action, obvious effect of inhibiting disease, and good overall tolerance of patients. However, they rarely restore organ function or reverse disability.

Mesenchymal stem cells (MSCs) are derived from the early developmental mesoderm and are multipotent stem cells with immunomodulatory and tissue repair functions [9]. MSCs were first discovered in bone marrow and were subsequently confirmed to be widespread in tissues such as adipose, umbilical cord, dental pulp, and hair follicles [10–12]. MSCs have the following characteristics: (1) low immunogenicity. MSCs do not express HLA-DR (human leukocyte antigen-DR) and costimulatory molecules involved in the activation of T and B lymphocytes (such as CD80, CD86, CD40), so they have lower immunogenicity [13–15]. Therefore, it is difficult to induce host immune rejection after allogeneic transplantation of MSCs. (2) Immunomodulatory properties. In the regulation of nonspecific immunity, MSCs can inhibit the differentiation and maturation of dendritic cells [16, 17], promote the polarization of macrophages toward the anti-inflammatory M2 phenotype[18–21], and inhibit natural killer cell proliferation [20, 22–24], thereby exerting anti-inflammatory and reducing antigen presentation effects. In addition, in the regulation of acquired immunity, MSCs can inhibit T-cell proliferation and induce T-cell apoptosis [25–27], promote the activation of regulatory T (Treg) cells [24, 28, 29], and induce proinflammatory Th1 cells to transform into anti-inflammatory Th2 cells [30, 31]. MSCs also interact directly with B cells, reducing plasmablast formation and promoting the induction of regulatory B cells [32, 33]. In addition, MSCs inhibit the proliferation of B lymphocytes by secreting soluble factors such as TNF-α, TGF-β, IFN-γ, and IL-10, reduce the secretion of antibodies, and simultaneously inhibit the expression of chemotactic receptors [34]. (3) Multipotential differentiation potential. MSCs have strong multidirectional differentiation potential and proliferation ability and thus have the function of repairing damaged tissues [12, 35]. For example, MSCs can differentiate into osteoblasts and chondrocytes under suitable in vivo or in vitro environmental conditions [36, 37].

MSCs can inhibit the host's immune rejection, regulate the body's immune system to exert anti-inflammatory effects and reduce antigen presentation, and undergo multidirectional differentiation to repair damaged tissues, so they have great application prospects in autoimmune diseases. Many preclinical and clinical studies have demonstrated the therapeutic effect of MSCs [9, 38, 39], but their clinical application also faces huge problems and challenges. For example, it is necessary to improve the homing of MSCs to the target site and the survival rate of MSCs in the host to ensure that MSCs can effectively play the role of immune regulation and tissue repair in the host for a long time. In addition, Joswig et al. found that immune rejection occurred after repeated injection of allogeneic MSCs at the same target. After the second intra-articular injection of MSCs into the horse model, significant adverse reactions occurred in the joint, including contamination and increased synovial assembly nucleating cell count [40]. Notably, MSCs are largely limited by their initial retention in pulmonary capillaries after intravenous injection, inconsistent expression of chemokine homing receptors, and lack of endothelial adhesion molecule expression [41–43]. This is further exacerbated by the inability to control cell fate in vivo, such as secretion of unwanted therapeutic factors, unpredictable engraftment and their differentiation into unwanted cell types in vivo.

MSCs can secrete extracellular vesicles (EVs) with lipid bilayer structures under resting or activated conditions, including exosomes (Exos), microvesicles and apoptotic bodies [44]. Among them, exosomes are the most important component in EVs, with a diameter of 30–150 nm, and are rich in cell-specific bioactive molecules, including lipids, proteins, microRNAs, and mRNAs. These active molecules are specifically selected and packaged by parental cells and enter the lipid bilayer, which is the key to stimulating the signal transduction mechanism in the body [45–47]. MSC-exos can mimic the immune regulation and tissue repair functions of parental MSCs. Compared with MSCs, the exosomes derived from them are more targeted, smaller in size, and can cross the blood–brain barrier to better exert immune regulation and anti-inflammatory functions [48]. However, there are still many challenges in the clinical application of MSC-exos: (1) Because the circulatory half-life of exosomes is short, it is always a challenge to provide therapeutic doses of exosomes to target sites. Systemic injection of exosomes has been proven to be rapidly cleared by blood circulation. (2) Different storage conditions also affect the activity of exosomes. After repeated freezing and thawing at − 80 °C, exosomes partially aggregated or fused, reducing the total number of exosomes and active substances [49]. (3) The immunomodulatory effect of MSC-exos is related to inflammatory factors in the environment, and the inflammation of different patients may also affect their therapeutic effect. (4) There is heterogeneity between exosomes, and it is also crucial to regulate the process of MSC-produced exosomes and modify them to improve the consistency of exosomes in autoimmune disease applications.

## Technologies for engineering MSCs and MSC-exos

**Fig. 1:**
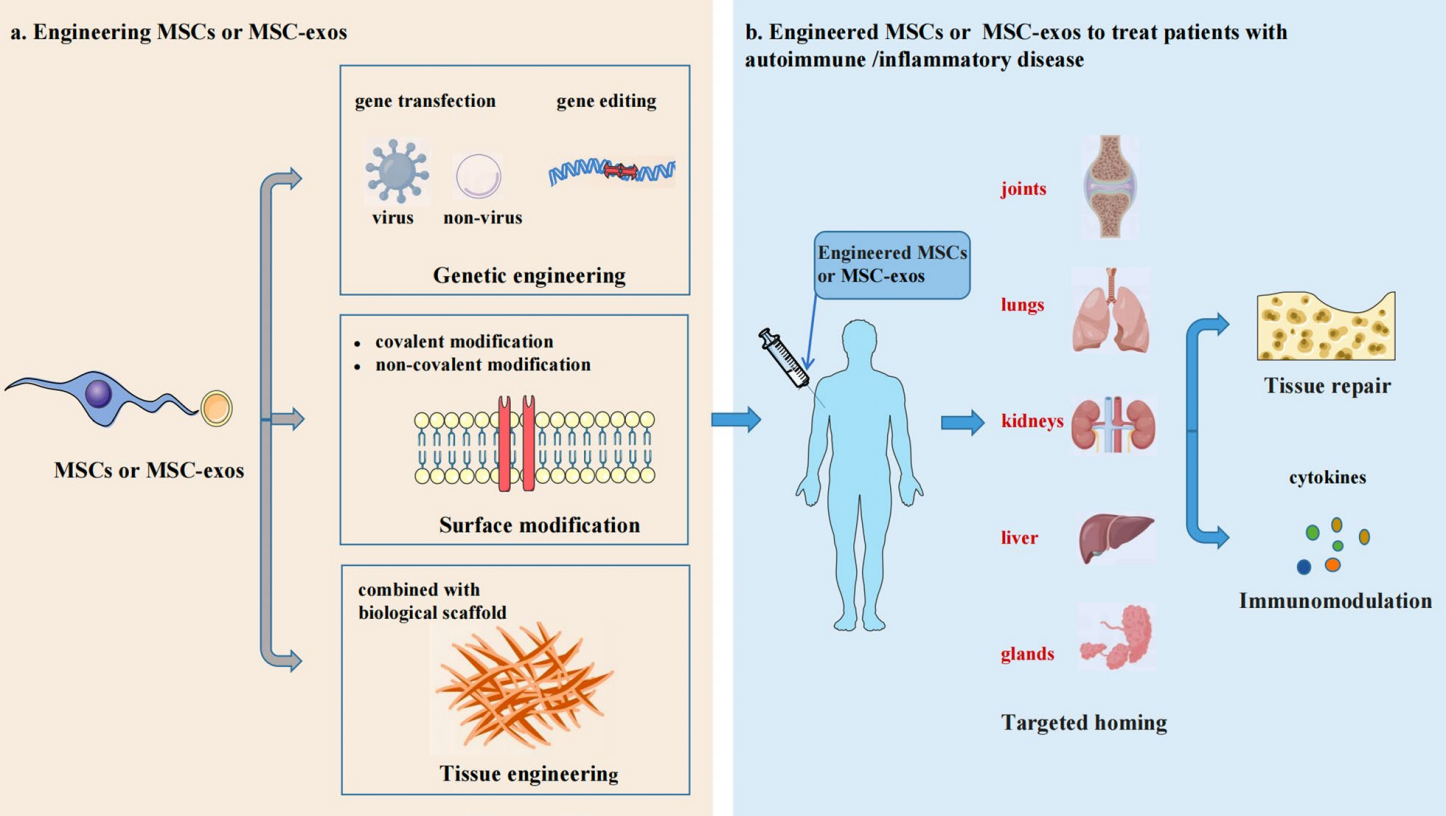
Technologies for engineering MSCs or MSC-exos. MSCs or MSC-exos can be modified by genetic engineering, surface modification and tissue engineering. Genetic engineering includes viral and nonviral methods. In addition, gene editing technology can also be used to achieve accurate editing of target genes. Surface modification includes covalent and noncovalent modifications. In addition, MSCs and MSC-exos can be modified with tissue engineering technology in combination with biological scaffold materials. In the treatment of autoimmune diseases, engineered MSCs or MSC-exos can further improve their targeted homing in patients to better play the role of tissue repair and immunomodulation. Created by myself

MSCs or MSC-exos can be modified through various techniques, such as genetic engineering, surface modification and tissue engineering, to enhance their targeted homing and therapeutic efficiency (Fig. 1).

### Genetic engineering

Genetic engineering of MSCs and MSC-exos is one of the ways to improve their therapeutic potential. Genetic modification of MSCs and MSC-exos to induce the expression of different proteins and soluble factors, such as growth factors, cytokines, transcription factors, chemokines, enzymes, and microRNAs, can improve their innate properties, such as survival, migration, and therapeutic potential. Genetic engineering includes both viral and nonviral methods.

#### Genetic modification of viral vector transfection

Most current preclinical and clinical applications of gene therapy use viral vector-mediated gene delivery. Viral vectors are generally characterized by high infectivity and ubiquity, but transfection efficiency may vary by target cell. MSCs are easily modified by viruses [50, 51]. Viral transfection ensures stable and long-term transcription of the gene of interest and is therefore more efficient than other methods for genetically engineering MSCs without the use of viral vectors [52]. Common viral vectors include retroviruses, lentiviruses, baculoviruses, adenoviruses, and adeno-associated viruses. Gene transfer using retroviral vectors can steadily integrate foreign genes into the host cell genome and permanently express the gene of interest [53]. However, the integration of retroviral vectors is random, with the potential danger of insertional mutagenesis and activation of oncogenes. Lentiviral vectors show better safety in the activation of proto-oncogenes after insertion into the host genome [53, 54], and for MSCs, which are difficult to transfect, the use of lentiviral vectors can greatly improve the target transfection efficiency of genes [55]. Interleukin-23 (IL-23) induces inflammation in autoimmune and inflammatory diseases by inhibiting Treg cells and promoting the response of Th17 cells and Th1 cells [56]. The soluble subtype of the IL-23 receptor can inhibit IL-23 signaling. Masoumeh et al. designed a recombinant IL-23 decoy receptor, cloned it into a lentiviral vector, and then transfected human adipose-derived MSCs (HAD-MSCs) with the lentiviral vector. The viral vector did not affect the properties of HAD-MSCs and simultaneously exerted the immunomodulatory effects of the IL-23 receptor and MSCs [57]. In addition, Hsiang-I et al. found that the receptors for the negative immune regulators PD-1 and LAG-3 were significantly elevated on T lymphocytes in clinical samples from kidney transplant patients and in heart transplant model mice. Therefore, they constructed PD-L1/FGL1 double-expressing MSC-exos by lentiviral vector, and both in vitro cell experiments and in vivo animal experiments confirmed that PD-L1/FGL1 double-expressing MSC-exos had stronger resistance to organ transplantation immune rejection [58]. Baculovirus is minimally virulent, neither replicates nor integrates into the host genome and is capable of transduction with high efficiency [59]. Baculoviruses can efficiently transduce MSCs [60, 61] without hindering their proliferative and differentiation potential. Since MSCs can differentiate into osteoblasts and bone morphogenetic protein-2 (BMP-2) is a potent osteogenic factor, Hu et al. transfected MSCs with a baculovirus vector expressing BMP-2 (Bac-CB), demonstrating that Bac-CB transfection directed the osteogenesis of naive MSCs. After implantation, transfected MSCs induced ectopic bone formation and promoted bone repair in the skull of nude mice [62]. Adenoviruses can transduce a variety of cell types, and their nonpathogenicity is their main advantage as gene transfer vectors. In addition, there is no risk of insertional mutagenesis, and the payload capacity of these vectors is high (~ 36 kb) [51, 63]. Nayak et al. transfected MSCs with an adenovirus vector to overexpress hepatocyte nuclear factor-4α and then injected the genetically modified MSCs into liver cirrhosis model mice through the tail vein. The results confirmed that MSCs can modulate anti-inflammatory function in mice to alleviate liver injury [64]. AAV is one of the most promising gene transfer vectors because of its low immunogenicity, good safety, wide host cell range, and long-term expression of foreign genes [65, 66]. However, AAV vectors still suffer from low transfection efficiency and host immune responses to AAV-transfected cells [63, 67].

To circumvent the problem of activating oncogenes and achieve targeted integration, researchers combined a nonintegrating lentiviral vector with zinc finger endonuclease (ZFN) technology. They used ZFN to insert the erythropoietin gene into the chemokine receptor-5 gene site of MSCs, making up for the shortcomings of lentivirus vectors in the application of gene site knockout and knock in [68]. Gene knockout or knock-in at specific sites in the target genome can also be achieved with tools such as transcription activator-like effector nucleases (TALENS) or clustered regularly interspaced short palindromic repeats (CRISPR/Cas9), which are Nucleases that recognize and direct gene integration in a site-specific manner [50]. Among them, CRISPR/Cas9 is the third-generation gene editing technology after the introduction of ZFN, TALENs and other gene editing technologies. It is one of the most efficient, easiest and lowest cost technologies in existing gene editing and gene modification. It has been used to transform MSCs [69–71]. Freitas et al. used CRISPR-Cas9 to edit the gene of MSCs to overexpress bone morphogenetic protein-9 (BMP-9). The results showed that overexpression of BMP-9 increased the osteogenic potential of MSCs. Then, after the edited MSCs were injected into the bone defect of rats, bone formation and bone density increased [72]. Similarly, Meng et al. used CRISPR-Cas9 to edit bone marrow-derived MSCs (BM-MSCs) to overexpress IL-10 and injected the edited BM-MSCs into diabetic myocardial infarction mice. The results showed that it inhibited the infiltration of inflammatory cells and the production of proinflammatory cytokines in mice, improved the recovery of cardiac function, alleviated cardiac injury, and increased angiogenesis [71]. Furthermore, based on the need for efficient cargo loading of EVs as biodelivery vehicles in therapeutic applications, Osteikoetxea et al. reversibly fused Cas9 with the sorting proteins of EVs through a protein heterodimerization system to package CRISPR/Cas9 into EVs, enabling precise editing of target genes and disease treatment [73].

#### Genetic modification for nonviral transfection

Although viral transfection is highly efficient, high production costs and adverse immune responses hinder viral transduction into the clinic [74]. In contrast, nonviral vectors are easy to scale up, have low immunogenicity and have a wide range of design options. Current methods for transfecting MSCs include electroporation and the use of liposomes, polymeric carriers and inorganic nanoparticles [75–79].

The electroporation transfection method utilizes a high voltage pulsed electric field to act on the cell membrane to reversibly perforate the cell membrane and introduce the target gene into MSCs. It produces stable transfectants with high frequency and transient gene expression efficiency, and it is simpler than other techniques [78, 80]. At present, the Nucleofection™ platform integrates traditional electroporation technology and cell-specific nucleofection solution to directly transfect exogenous genes into the nucleus, realizing the high efficiency of primary cells that have been troubled by the restriction of cell division. Transfection, which has been successfully applied to transfect MSCs, may potentially affect the innate capacity of MSCs [81]. To this end, Jae et al. established an electroporation gene delivery system optimized for genetic modification of placenta-derived MSCs (PD-MSCs) [82]. PD-MSCs were transfected with nonviral AMAXA electroporation technology to overexpress the PRL-1 plasmid gene, and the lentivirus system was used as its positive control group. The results showed that the mRNA expression of PD-MSCs overexpressing PRL-1 was enhanced by both gene delivery systems. The nonviral electroporation system transfected PD-MSCs PRL-1 was as effective as the lentiviral system and safer than the lentiviral system. Therefore, this nonviral electroporation technique may become a new strategy for the clinical application of stem cell therapy.

Liposomes and polymers are popular as nonviral vectors and have made significant progress in delivering various types of genetic material for the treatment of various diseases [83], but their efficiency in transfecting MSCs is low. For example, with commercial Lipofectamine 2000 optimized for the transfection of MSCs, only 10–30% of cells were successfully transfected [84–86], while the commonly used 25 kDa branched polyethyleneimine can only achieve approximately 20% transfection efficiency [85, 87]. In addition, transfection of MSCs with liposomes and polymeric vectors is significantly toxic, limiting transgene expression levels and their therapeutic efficacy [88, 89]. To this end, Hamann et al. conducted a study and found that in human MSCs (hMSCs) transfected with plasmid DNA liposomes, prestimulating cells with glucocorticoids could significantly increase the transfection rate of hMSCs and prolong the transgene duration of expression [90]. Glucocorticoids regulate the expression of endogenous hMSCs genes by activating the cytoplasmic glucocorticoid receptor to improve their oxidative stress, apoptosis and inflammatory responses and prevent the decline in their metabolism and protein synthesis, resulting in enhanced expression of their transgenes.

Inorganic nanoparticles, mainly combined with polycations, have also been used to transfect MSCs. Gold nanoparticles modified with Jet-PEI reagent can condense DNA on the surface, resulting in a 2.5-fold increase in transfection efficiency over conventional Jet-PEI multimers [76]. Muroski et al. prepared Ku70 peptide-modified gold nanoparticles with N-cysteine, which were able to transfect rat MSCs with a transfection rate of nearly 80% without affecting the activity and performance of MSCs. [91].

### Surface modification

Surface modification techniques that bind to proteins overexpressed in diseased or damaged tissues through covalent or noncovalent interactions can increase the number of MSCs transported to the target and potentially enhance subsequent therapeutic effects. Furthermore, these methods of modifying MSCs can enhance the adhesion and engraftment of MSCs without significantly affecting the viability, pluripotency and differentiation potential of the cells [92, 93]. Surface modification techniques include covalent modification (such as enzymatic and chemical modification) and noncovalent modification.

Enzymatic modifications are limited to glycoproteins already present on the cell surface, potentially affecting other key cell surface molecules through nonspecific interactions [94]. Chemical modifications enable MSCs to provide several targeting moieties by covalent coupling methods, including ligands [95], peptides [92] and polymers [96]. Noncovalent modifications can improve cell-host properties without compromising the native properties of MSCs [97].

Currently, D'Souza et al. combined a covalent coupling approach with polymer-based engineering to enhance the homing properties of MSCs for the treatment of bone injury sites [96]. They synthesized bone-targeting moieties containing alendronate using atom transfer radical polymerization. This drug has a high affinity for bone and can slow bone resorption by inducing osteoclast apoptosis [98]. The results demonstrated that MSCs carrying bone-targeting polymers could form hydroxyapatite crystals and rodent bone fragments for bone regeneration, as well as repair of inflammatory tissues.

Furthermore, Lathwal et al. combined a noncovalent approach with polymer-based engineering to combine exosomes with functional polymers. They synthesized exosome-polymer hybrids (EPH) from MSC-exos, curcumin-loaded macrophage-derived exosomes and BMP-2-loaded macrophage-derived exosomes and evaluated the angiogenic capacity of EPH [99]. It was found that in human umbilical vein endothelial cells and lymphatic endothelial cells, MSC-exos maintained their angiogenic potential and induced early angiogenesis even after polymer conjugation. Similarly, BMP-2-exosomes and curcumin-exosomes also retained their biological activities to induce osteoblast differentiation and downregulate NF-κB, respectively. This EPH could precisely control the interactions on the surface of exosomes by modulating the length and surface loading of the polymer while retaining their inherent biological activity. In addition, it was more stable than native exosomes under different storage conditions. In summary, these properties overcome some of the major limitations of the application of exosomes as drug delivery systems.

### Tissue engineering

Tissue defects or dysfunctions caused by various reasons are the main reasons for endangering human life and health. Traditional therapies such as tissue transplantation and prosthetic replacement suffer from disadvantages such as tissue shortages, disease transmission, and biocompatibility issues [100]. Tissue engineering can avoid the traditional treatment mode of "repairing wounds with wounds", showing broad application prospects. Seed cells, biological scaffolds and tissue construction are the three most basic elements of tissue engineering. As a source of seed cells for tissue engineering, the combined use of MSCs and MSC-exos with biological scaffolds is more conducive to exerting their advantages and has broad application prospects in tissue repair and reconstruction.

Due to their good biocompatibility, ductility and thermal stability, the nanotopological scaffolds formed by composite materials provide good compatible conditions for the adhesion and proliferation of seed cells [101]. Nanotopology can guide the proliferation, differentiation and expression of specific functional genes of MSCs [102, 103]. Most nanotopologies tend to guide the differentiation of MSCs in a single direction, and some nanotopologies have broad effects. For example, nanoisland structures formed by polymer composites can not only induce MSCs to differentiate into osteoblasts, cartilage and fat but also effectively stimulate cell proliferation and promote tissue regeneration [104]. Most of the findings indicate that various nanotopologies have positive effects on the growth and differentiation of MSCs. However, there is also evidence that certain types of nanotopology may have adverse effects on the body. For example, nanogrooves are not conducive to the differentiation of MSCs into chondrocytes, nor are they suitable for cartilage repair or regeneration [105, 106]. In addition, over time in the implant, the nanomaterial corrodes and releases nanoparticles near the implant. Due to the surface properties of these particles, macromolecules in their vicinity can be adsorbed, resulting in changes in the surface properties of nanoparticles, with toxic side effects on cell morphology, adhesion, proliferation and differentiation [102].

Biomedical hydrogels, such as hyaluronic acid, chitosan, and polyethylene glycol, are structurally similar to natural extracellular matrices with good hydrophilicity, biocompatibility, biodegradability and encapsulation ability. Polymer hydrogels can provide a hydrating environment for MSCs and promote their proliferation and differentiation [107–109], and the 3D network formed by their cross-linking can swell through water absorption to fill in tissue defects and provide scaffolding for cells [110–113]. In addition, hydrogel crosslinkers should be biodegradable by hydrolysis or enzymatic degradation to replace the hydrogel with extracellular matrix as tissue regeneration progresses [114]. However, these systems are mainly biologically inert, so further improvement is required to achieve tissue-specific biological activity [114, 115]. Methods for introducing bioactivity into hydrogels include providing tissue-specific growth factors, polypeptides, and polysaccharide-like bioactive macromolecules, which involve reagents or side reactions that are toxic to cells and may compromise the biocompatibility of the material [116].

Recently, Guo et al. designed a hydrogel crosslinker with tissue-specific functionalized PdBT, which in the presence of catalyst can be achieved by simply stirring the corresponding components in room temperature water. Binding of hydrogels to related polypeptides and macromolecular bioactive molecules [117]. In addition, this study also utilized a PdBT crosslinking agent for P(NIPAAm-co-GMA) hydrogel crosslinking, which successfully achieved cytocompatibility, rapid crosslinking and hydrolytic degradation, and this crosslinking agent was also suitable for MSCs. hydrogel encapsulation system [117].

Furthermore, based on the fact that the hydrogel cannot maintain its shape, a scaffold composed of special materials can be used to form a hybrid composite material with high cytocompatibility and good mechanical properties. Zhang et al. used 3D printing to fabricate custom nano-hydroxyapatite/poly-ε-caprolactone (nHP) scaffolds in a rat calvarial defect model using umbilical cord MSC-derived exosomes (UCMSC-exos) encapsulated injectable hyaluronic acid hydrogels that completely filled the pore structure of nHP scaffolds, and it was found that these engineered UCMSC-exos could promote cranial defect repair in vivo and had a good proangiogenic effect in vitro [118]. In addition, based on Neural EGFL-like 1 (Nell1) as an exocrine protein associated with craniosynostosis to promote the osteogenic differentiation of BMSCs, Lan et al. modified BMSC-derived cells with Nell1 gene extracellular vesicles (Nell1/EVs), constructing an extracellular vesicle-hydrogel composite system (3D-Nell1/EV-hydrogel system) [119]. The results confirmed that Nell1/EVs could induce stem cells to differentiate into osteoblasts by downregulating miR-25-5p, which can inhibit osteogenesis by targeting Smad2 and inhibiting the activation of the SMAD and ERK signaling pathways. In addition, the 3D-Nell1/EV-hydrogel system could realize the slow and sustained release of EVs in the bone defect area and the preservation of high concentrations, which can effectively promote the repair of large-scale bone defects in animals [119].

## Application of engineered MSCs in autoimmune diseases

### RA

RA is a common systemic autoimmune disease that mainly affects the small joints of the whole body. The main pathological features are synovial hyperplasia, inflammatory cell infiltration and synovial pannus formation. Treatment options for RA are limited, providing immunomodulation and articular cartilage-bone regeneration in damaged joints [120].

Studies in collagen-induced arthritis (CIA) models showed that transgenic MSCs expressing human soluble tumor necrosis factor receptor 2 (HsTNFR2) partially prevent arthritis symptoms and produce better therapeutic effects than MSCs alone [121]. In addition to the inherent anti-inflammatory effects of MSCs, the expression of HsTNFR2 could bind and deceive TNFα, blocking TNF-α-mediated inflammatory effects. MSCs expressing hepatocyte growth factor (HGF) alleviated arthritis symptoms in CIA mice, but in the late stages of disease, the effect was no better than treatment with MSCs alone [122]. This was due to the immunosuppressive effect of HGF in the early stage of the disease, but in the late stage, HGF could induce the activation of fibroblast-like synoviocytes (Fls), which can produce IL-6 to induce inflammation and cell proliferation and reduce apoptosis.

Etanercept is a TNF-α blocker currently used to treat RA. Narae et al. constructed a microcircular plasmid loaded with etanercept (mcTNFR2) and used electroporation to transfect MSCs [123]. The generated mcTNFR2 MSCs successfully produced the expected etanercept, and CIA mice injected with mcTNFR2 MSCs improved arthritis symptoms more effectively than mice injected with conventional MSCs or etanercept alone. Compared with nonengineered MSCs, engineered MSCs have more advantages in inhibiting arthritis due to the secretion of etanercept.

In RA, IL-17 induces synovial fibroblasts, macrophages and chondrocytes to produce proinflammatory mediators such as IL-1 and TNF-α. Kim et al. epigenetically (Epi) modified hMSCs with hypomethylating agents or HDAC inhibitors and intervened in synovial fluid mononuclear cells (SFMCs) from RA patients. The results showed that after treatment with Epi-hMSCs, the levels of IL-17 and IFN-γ secreted by SFMCs were significantly reduced, and they also had a greater immunosuppressive effect on T-cell proliferation, cytokine expression and Th17 cell differentiation [124]. Notably, this study found that epigenetic modification can enhance the function of hMSCs in in vitro experiments, but its efficacy and safety need to be studied in animal models in the future.

Recently, Gang et al. successfully combined thermochemotherapy with tissue engineering to eliminate inflammation and regenerate RA-induced cartilage defects [125]. They loaded the anti-rheumatic drug methotrexate (MTX) and transforming growth factor β1 (TGFβ1) into the multifunctional double network hydrogel constructed by nano-Fe_3_O_4_ composite chitosan polyolefin (DN-Fe-MTX-TGFβ1). Among them, the mechanical properties of the hydrogel were comparable to those of articular cartilage, ensuring its stability as a scaffold. Moreover, the long-term release ability and magnetocaloric properties of the hydrogel ensured that it provided sustained localized thermochemotherapy. The results showed that in the DN-Fe-MTX-TGFβ1 hydrogel, the activation of macrophages was significantly inhibited with a good anti-inflammatory effect. At the same time, the chondrogenic differentiation of MSCs was significantly improved, which could promote the repair of cartilage defects.

### SLE

SLE is a chronic autoimmune disease with clinical manifestations of multisystem damage [126, 127]. Its pathogenesis depends on loss of tolerance and sustained production of autoantibodies, characterized by the presence of autoantibody-producing autoreactive T cells and hyperactive B cells [128–130]. These cells form deposits of immune complexes that destroy different tissues that express self-antigens.

IL-37 is a member of the IL-1 family with immunosuppressive activity, and previous studies have shown that IL-37 can inhibit the expression of inflammatory factors in peripheral blood mononuclear cells of SLE patients [131]. Xu et al. used lentiviral vectors to transfect MSCs to overexpress IL-37 and evaluated the effect of IL-37-MSCs on immunosuppression in vitro. Then, these cells were transplanted into MRL/LPR mice (SLE model) [132]. Compared with the control group, mice transplanted with IL-37-MSCs had improved survival and reduced systemic lupus erythematosus symptoms, proinflammatory factors (IL-1β, TNF-α, IL-17 and IL- 6) expression, total and autoantibodies (anti-dsDNA and anti-ANA) in serum and urine as well as T-cell numbers in serum and kidney.

### OA

Osteoarthritis (OA) is a chronic degenerative joint disease affecting approximately 15% of the global population [133, 134]. OA is characterized by bone loss, articular cartilage degeneration, articular margin osteophyte formation, and subchondral bone hyperplasia. The pathogenesis of OA involves chronic low-grade inflammation that severely hinders chondrocyte proliferation and cartilage matrix deposition [135, 136]. Various immune cells have been identified in the synovium of OA patients. Of these, macrophages, T cells and B cells are the most abundant [136, 137].

To improve the efficacy of MSCs for the treatment of OA, the Viswanathan lab pioneered an engineering strategy for culturing MSCs in 3D aggregates. The results showed that 3D MSCs reduced inflammation, fibrosis and cartilage degradation in both in vitro and in vivo models of OA compared to MSCs cultured using conventional 2D methods [138]. Furthermore, Brian et al. isolated MSCs from the knee joint synovial fluid of three OA patients, epigenetically reprogrammed them, and then induced differentiation to establish a reprogrammed MSC (Re-MSC) line. Compared with MSCs, Re-MSC increased in vitro proliferative capacity and improved articular chondrocyte differentiation capacity, promoting articular cartilage repair in an animal model of spontaneous OA [139].

In addition, innovative tissue engineering approaches using allogeneic MSCs are currently available to exploit the innate chondrogenesis potential of MSCs, namely, the development of tailored three-dimensional (3D), direct and immediate replacement of hyaline cartilage, promoting cells to a stable hyaline-like shape in vitro. Cartilage transition improves tissue integration at local sites and optimizes treatment outcomes [140]. At the same time, MSCs can be engineered using CRISPR or other gene editing techniques to further enhance the cartilage potential and robust tissue regeneration and integration of MSCs.

### IBD

IBD is a group of chronic intestinal inflammatory diseases, including Crohn's disease and ulcerative colitis. IBD is mainly related to genetic susceptibility, environmental factors, and autoimmunity [141, 142]. The current main therapeutic approach is to control the inflammatory response and modulate the body's immune function [143, 144].

Intercellular adhesion molecule-1 (ICAM-1) is involved in signal transmission between cells and plays an important role in regulating the body's immune response [145]. When an inflammatory reaction occurs, MSCs can significantly upregulate the expression of ICAM-1. Upregulation of ICAM-1 expression helps to enhance the immunosuppressive effect of MSCs [146]. A study showed that genetically engineered MSCs overexpressing ICAM-1 could alleviate pathological damage to colon tissue, improve the general condition of IBD mice, promote body weight recovery, and reduce the mortality rate of mice. Compared with other treatment groups, ICAM-1- MSCs significantly reduced the number of Th1 and Th17 cells while increasing the proportion of Treg cells [147]. The results of this study suggested that the overexpression of ICAM-1 could enhance the ability of MSCs to regulate Th cell subsets, promote the migration of MSCs to damaged tissues and reduce the inflammatory response locally, thereby playing a better therapeutic role in IBD.

IL-25 inhibits the differentiation of IBD CD4^+^ T cells into Th1/Th17 cells, thereby reducing various inflammatory lesions [148]. The expression of IL-25 is significantly decreased in the damaged mucosa of IBD. It was found that IL-25-induced MSCs had an enhanced ability to inhibit Th17 cell differentiation in rats with colitis while significantly increasing the number of T cells [149]. In addition, IL-25-induced MSCs could also promote the proliferation and migration of intestinal epithelial cells [150].

By upregulating cell adhesion molecules such as ICAM-1 [151], vascular cell adhesion molecule-1 (VCAM-1) and mucosal addressin cell adhesion molecule (MAdCAM) [152], MSCs can be localized to the site of inflammation to play a role. In vitro, ICAM-1-coated MSCs significantly enhanced their adhesion to activated endothelial cells [151]. In an in vivo model of inflammatory bowel disease, the survival of the treatment group injected with MAdCAM-coated MSCs was prolonged (approximately 2–3 times that of unmodified MSCs), demonstrating that MAdCAM or VCAM-1 guides the homing ability of MSCs. VCAM-1-coated MSCs could efficiently localize to the colon and suppress Treg cells in vivo [152].

## Application of engineered MSC-exos in autoimmune diseases

### RA

Among the cells involved in RA, M1 macrophages are known as the most prominent cells responsible for the formation and progression of lesions by releasing various types of proinflammatory cytokines, such as TNF-α, IL-6 and IL-1 [153, 154]. MSC-exos induce macrophage polarization from the M1 to M2 phenotype in inflamed tissue [155], but their poor biodistribution in vivo compromises therapeutic efficacy [156, 157]. To this end, Dong et al. used metabolic glycoengineering of adipose-derived stem cells to surface-modify MSC-exos to target activated macrophages in RA-inflamed joints [158]. Studies have demonstrated that surface-modified MSC-exos introduce a broad range of targeting moieties without functional impairment of their structural or functional cargo. Furthermore, following systemic administration to CIA mice, engineered MSC-exos promoted the polarization of macrophages from M1 to M2 in their inflamed sites and decreased peripheral proinflammatory cells (M1 macrophages, activated synovial fibroblasts).

In the CIA mouse model, researchers established a biomimetic exosome and modified its surface with folic acid (FA)-polyethylene glycol (PEG)-cholesterol (Chol) compound to obtain FPC-Exo/Dex Active Targeted Drug Delivery Systems [159]. The results showed that the FPC-Exo/Dex system downregulated the levels of proinflammatory cytokines and upregulated the levels of anti-inflammatory cytokines and could better protect the bone and cartilage of CIA mice and significantly reduce joint inflammation. In addition, the bionic drug delivery system had no obvious liver toxicity and good biocompatibility.

### OA

The wet tissue environment of articular cartilage in OA patients puts forwards higher requirements for the adhesion properties of the hydrogel itself. Zhang et al. prepared hydrogels with high binding strength to wet surfaces using a cross-linked network composed of alginate-dopamine (AD), chondroitin sulfate (CS) and regenerated silk fibroin (RSF). Compared to commercial embedded tissue adhesives, AD/CS/RSF hydrogels provided a relative lap shear strength of 120 kPa with a similar gel time and a higher ability to maintain bond strength [160]. After the hydrogel was encapsulated with BMSC-exos, it was injected into the knee articular cartilage defect of rats, and it was found that the AD/CS/RSF/EXO hydrogel encapsulated with BMSC-exos could promote the recruitment of BMSCs and promote BMSCs proliferation and differentiation into chondrocytes, as well as in situ regeneration of cartilage defects and cartilage remodeling. The exosomes released from the hydrogel could also recruit BMSCs in situ around the hydrogel and new cartilage through chemokine-related signaling pathways.

To improve the intra-articular bioavailability of MSC-EVs for OA treatment, Feng et al. developed a strategy to modify MSC-EVs with a novel cationic amphiphilic macromolecule (ε-polylysine-polyethylene-distearyl phosphatidylethanolamine, PPD). Positively charged MSC-EVs were obtained by incubation with 100 μg/ml PPD. The modification process hardly interfered with the integrity and content of MSC-EVs and showed good stability under the interference of anionic macromolecules. The findings suggested that the surface charge of MSC-EVs could be effectively reversed from electronegative to electropositive through PPD modification to increase their absorption, penetration and retention in cartilage, ultimately enhancing the treatment of OA [161].

### IBD

The programmed cell death protein-1/programmed cell death protein-L1 (PD-1/PD-L1) signaling pathway plays an important role in suppressing the initial and effector phases of immune responses and maintaining immune homeostasis [162]. Recently, Xu et al. constructed MSC-EVs with high PD-L1 expression using a lentiviral vector, which could initiate immunosuppressive signals by interacting with PD-1 in activated immune cells to maintain immune tolerance. By constructing a mouse model of ulcerative colitis, it was found that this PD-L1-expressing extracellular vesicle could specifically target the lesion and remodel the normal physiological function of the lesion by regulating the immune microenvironment [163].

Recently, therapeutic mRNA delivery in EVs has been challenged by low loading efficiency. Zhang et al. designed a DNA aptamer consisting of two parts: the single-stranded part recognizes the AUG region of the target mRNA and blocks mRNA translation and ribosome assembly, and a double-stranded portion contains elements recognized by CD9-ZF (zinc finger) motifs, sorting mRNA of DNA aptamers into CD9-ZF-engineered EVs. In vitro and in vivo studies have shown that this system could efficiently load functional mRNA into EVs and that fat-specific delivery of Pgc1α mRNA loaded by this strategy could effectively induce white adipocyte browning [164]. Furthermore, IL-10 mRNA delivered by this strategy had potent anti-inflammatory effects in a mouse model of IBD [164].

## Challenges in transferring engineered MSCs and MSC-exos from bench to bed-side

Through genetic engineering, surface modification and tissue engineering, MSCs or MSC-exos are engineered to further improve their targeting and other biological characteristics. However, there are some shortcomings and limitations in its clinical application.

First, the technology of engineering MSCs or MSC-exos itself has certain limitations. (1) In gene transformation, although the efficiency of virus transfection is very high, the high production cost and adverse immune reaction hinder the clinical application of virus transduction. On the other hand, nonviral gene therapy is severely limited due to low transfection efficiency. It is necessary to optimize all aspects of nonviral gene transfer, such as constructing efficient plasmids and improving the transfection scheme. (2) Although studies on the surface modification of MSCs or MSC-exos have not found that their functions are seriously impaired, potential risks still exist before long-term and detailed in vivo studies. Since homing and treatment are coordinated by multiple effector molecules, improving the target location of modified MSCs can neither ensure enhanced MSC migration nor ensure higher treatment results [152]. (3) In addition, nanomaterials, which play a role as scaffolds in tissue engineering, corrode with the prolongation of implant time and release nanoparticles near the implant, causing toxic and side effects on cell morphology, adhesion, proliferation and differentiation [102]. At the same time, the uneven distribution of oxygen and nutrients in the 3D spatial structure will also affect stem cells.

Second, the treatment of autoimmune/inflammatory diseases by engineered MSCs or MSC-exos is still in animal and preclinical studies. Due to serious legal barriers, some types of engineered MSCs or MSC-exos are now difficult to translate into clinical practice. Therefore, it is necessary to conduct strict in vitro tests under clinically relevant conditions, as well as tests in large samples, to evaluate their effects in vivo. In addition, the key problem of applying engineered MSCs or MSC-exos to clinical practice is how to ensure the safety of engineering transformation and how to standardize the final treatment product.

## Conclusion and outlook

MSCs have pluripotent differentiation potential, low immunogenicity, and immunomodulatory properties and have been widely used in autoimmune diseases. MSC-exos have similar functions to MSCs. However, their therapeutic efficacy in vivo is compromised by their homing ability. For example, inconsistent and insufficient homing of MSCs and MSC-exos to target tissues after systemic infusion is considered to be the main reason for insufficient efficacy. The engineering of MSCs and MSC-exos by various technical means provides new approaches for further improving their targeting and therapeutic efficiency (Table 1) and shows great potential in the treatment of autoimmune diseases (Table 2). Although engineering approaches can overcome many of these obstacles, their clinical application also has many challenges. The technology of engineering MSCs or MSC-exos needs to be further optimized to ensure the safety and effectiveness of their engineering transformation. In addition, the engineering strategy needs to be tested strictly in vitro under clinically relevant conditions and in large samples to evaluate its impact in vivo.

**Table 1:** Technologies for Engineered MSCs and Exos

Engineering technology	Advantages	Disadvantages	References
*Genetic engineering*			
Retrovirus	DNA integration into the host cell genome	Insertional mutation	[54]
	Long-term stable expression of target gene	Oncogene activation	
Lentivirus	DNA integration into the host cell genome	Oncogene activation	[54–56]
	Long-term stable expression of target gene		
	Infects dividing and quiescent cells		
	No oncogene inserted		
Baculovirus	Low toxicity	Oncogene activation	[60–62]
	High transfection efficiency		
	Does not affect cell function		
Adenovirus	Non-pathogenic High load capacity	Oncogene activation	[52, 64]
		Transient gene expression	
Adeno-associated virus	Low immunogenicity	Low transfection efficiency	[64, 66–68]
	high security	Oncogene activation	
	Long-term expression of target gene		
Electroporation	High transfection efficiency	May affect cell function	[78, 80, 81]
	Simple operation		
Liposomes and Polymers	Easy to synthesize and modify	Low transfection efficiency	[84–86, 88, 89]
		Cytotoxicity	
Inorganic Nanoparticles	Easy to synthesize and modify	Cytotoxicity	[75, 91]
	Good biocompatibility		
*Surface modification*			
Enzyme modification	Enhances cell adhesion and engraftment	Nonspecific	[92–94]
	Minimal impact on cell viability and function	Potentially affects other cell surface molecules	
chemical modification	Strong targeting		[92, 95, 96]
	Does not affect other cell surface molecules		
Non-covalent modification	Strong targeting		[97]
	Does not affect other cell surface molecules		
*Tissue engineering*			
Nanotopological scaffold	Good biocompatibility Strong ductility Good thermal stability	Corrosion occurs when implanted in the body for too long	[101, 102]
Hydrogel scaffold	Good biocompatibility		[107–109]
	Strong Hydrophilicity		
	Good biodegradability Strong encapsulation ability

**Table 2:** Application of engineered MSCs and exosomes in autoimmune diseases

Disease	Cells/exosomes	Engineering technology	Results	References
RA	MSCs	Genetically modified MSCs to overexpress HsTNFR2	Prevented arthritis symptoms	[121]
	MSCs	Genetically modified MSCs to overexpress HGF	Alleviated joint inflammation in CIA mice	[122]
	MSCs	Gene transfer of etanercept-loaded micro circular plasmids to MSCs by electroporation	Improved arthritis symptoms in CIA mice	[123]
	hMSCs	Epigenetic modification of hMSCs to enhance cellular function	Enhanced immune suppression	[124]
	MSCs	MTX- and TGFβ1-loaded hydrogels combine with MSCs	Anti-inflammatory	[125]
			Promoted cartilage differentiation of MSCs	
	MSC-Exos	Surface modification of MSC-Exos by metabolic glycoengineering	Promoted polarization of macrophages and reduced the activity of pro-inflammatory cells at the inflammatory site in CIA mice	[158]
	Exos	Surface modification of MSC-Exos to establish targeted drug delivery system	Alleviated joint inflammation in CIA mice	[159]
SLE	MSCs	Lentiviral transfection of MSCs to overexpress IL-37	Inhibition of inflammatory factors	[132]
			Relieved symptoms in lupus model mice	
OA	MSCs	3D culture of MSCs	Reduced inflammation, fibrosis, and cartilage degradation in vivo and in vitro	[138]
	MSCs	Epigenetically reprogrammed MSCs	Increased in vitro proliferation ability and improve articular chondrocyte differentiation	[139]
	BMSC-Exos	Hydrophilic Hydrogel combined with Exos	Promoted cartilage regeneration and remodeling	[160]
	MSC-EVs	MSC-EVs modified by PPD to changes its surface charge	Increased absorption, penetration and retention in cartilage	[161]
IBD	MSCs	Genetically modified MSCs to overexpress ICAM-1	Enhance immune regulation and reduce colon tissue damage	[147]
	MSCs	ICAM-1-coated MSCs	Promotes adhesion to endothelial cells	[151]
	MSCs	VCAM-1-coated MSCs	Prolonged survival in IBD mice	[152]
	MSC-EVs	Lentiviral transfection of MSC-EVs to overexpress PD-1	Targeted lesions for immune regulation	[163]
	EVs	Designed a DNA aptamer to enhance EVs mRNA delivery	Efficiently induced browning of white adipocytes	[164]
			Promoted anti-inflammatory in IBD mice	

## Data Availability

Not applicable.

## Abbreviations

MSCs: Mesenchymal stem cells
Exos: Exosomes
MSC-exos: MSCs-derived exosomes
RA: Rheumatoid arthritis
SLE: Systemic lupus erythematosus
OA: Osteoarthritis
IBD: Inflammatory bowel disease;
HLA-DR: Human leukocyte antigen-DR
TNF: Tumor necrosis factor
Tregs: Regulatory T cells
EVs: Extracellular vesicles
AAV: Adeno-associated virus
IL-23: Interleukin-23
HAD-MSCs: Human adipose-derived MSCs
BMP-2: Bone morphogenetic protein-2
Bac-CB: Baculovirus vector expressing BMP-2
ZFN: Zinc finger endonuclease
TALENS: Transcription activator-like effector nucleases
CRISPR/Cas9: Clustered regularly interspaced short palindromic repeats
BMP-9: Bone morphogenetic protein-9
BM-MSCs: Bone marrow-derived MSCs
PD-MSCs: Placenta-derived MSCs
hMSCs: Human MSCs
EPH: Exosome-polymer hybrids
nHP: Nano-hydroxyapatite/poly-ε-caprolactone
UCMSC-exos: Umbilical cord MSCs-derived exosomes
Nell1: Neural EGFL-like 1
CIA: Collagen-induced arthritis
HsTNFR2: Human-soluble tumor necrosis factor receptor 2
HGF: Hepatocyte growth factor
mcTNFR2: Micro-circular plasmid loaded with etanercept
Epi: Epigenetically
SFMCs: Synovial fluid mononuclear cells
MTX: Methotrexate
TGFβ1: Transforming growth factor β1
Re-MSCs: Reprogrammed MSCs
3D: Three-dimensional
ICAM-1: Intercellular adhesion molecule-1
VCAM-1: Vascular cell adhesion molecule-1
MAdCAM: Mucosal addressin cell adhesion molecule
FA: Folic acid
PEG: Polyethylene glycol
Chol: Cholesterol
AD: Alginate-dopamine
CS: Chondroitin sulfate
RSF: Regenerated silk fibroin
PPD: ε-Polylysine-polyethylene-distearyl phosphatidylethanolamine
PD-1: Programmed cell death protein-1
PD-L1: Programmed cell death protein-L1
